# HIV Prevalence and Impact on Renutrition in Children Hospitalised for Severe Malnutrition in Niger: An Argument for More Systematic Screening

**DOI:** 10.1371/journal.pone.0022787

**Published:** 2011-07-28

**Authors:** Yoann Madec, David Germanaud, Violeta Moya-Alvarez, Wafa Alkassoum, Aichatou Issa, Morou Amadou, Stephanie Tchiombiano, Cecilia Pizzocolo, Florence Huber, Sanata Diallo, Roubanatou Abdoulaye-Mamadou

**Affiliations:** 1 Unité d'Epidémiologie des maladies Emergentes, Institut Pasteur, Paris, France; 2 Solthis, Paris, France; 3 Service de pédiatrie, Hôpital national de Niamey, Niamey, Niger; 4 Solthis, Niamey, Niger; Boston University, United States of America

## Abstract

**Background:**

In developing countries, malnutrition is a contributing factor in over 50% of child deaths. Mortality rates are higher in underweight children, and HIV-infection is known to increase underweight. Our goals were to evaluate the prevalence of HIV among children hospitalised for severe malnutrition (SM) at the Niamey national hospital (Niger), and to compare renutrition and mortality by HIV-status.

**Methods:**

Retrospective study based on all children <5 years hospitalised for SM between January 1^st^ 2008 and July 1^st^ 2009. HIV-prevalence was the ratio of HIV+ children on the number of children tested. Duration of renutrition and mortality were described using survival curves.

**Results:**

During the study period, 477 children were hospitalised for SM. HIV testing was accepted in 470 (98.5%), of which 40 were HIV+ (HIV prevalence (95% confidence interval) of 8.6% (6.2–11.5)). Duration of renutrition was longer in HIV+ than HIV− children (mean: 22 vs. 15 days; p = 0.003). During renutrition, 8 (20%) and 61 (14%) HIV+ and HIV− children died, respectively (p = 0.81).

**Conclusion:**

Around 9% of children hospitalised for severe malnutrition were HIV infected, while in Niger HIV prevalence in adults is estimated at 0.8%. This pleads for wider access to HIV testing in this population.

## Introduction

In developing countries, malnutrition contributes to nearly 50% of deaths in children, although it may not be the direct cause of death [1]. In children, mortality is strongly related to body growth parameters represented by weight-for-height, weight-for-age and height-for-age z-scores. The risk of death was 10 fold higher in children with a weight-for-height z-score <−3 (severe wasting) when compared to children with a z-score higher or equal to −1 [2].

Malnutrition can be attributed to nutritional deficiency, still malnutrition is multi factorial and HIV-infection can also induce or aggravate it. In fact, wasting is a WHO stage 3 AIDS condition. This interaction between HIV infection and malnutrition is all the more important since areas of relatively high prevalence of both conditions often overlap in developing countries.

A study conducted in Malawi showed that mortality during renutrition was higher in HIV-positive children than in HIV-negative children [3], [4]. But this study took place in a context of very high HIV-prevalence. Furthermore, it is not known whether children infected with HIV respond differently to renutrition than uninfected children, particularly in terms of duration of renutrition.

Very few studies have been conducted to study how the burden of HIV affects malnutrition in developing countries, and little is known about nutritional and clinical recovery of HIV-positive children. In this study, we aimed at estimating HIV prevalence in children hospitalised for severe malnutrition in a context of low HIV prevalence, and at comparing the recovery during hospitalisation in terms of weight gain and mortality.

## Methods

### Ethics statements

The protocol of this study has been submitted and approved by the Niger ethics committee (Comité Consultatif National d'Ethique). Patients' consent was not asked for as the study was retrospective and data was entered in the database and analysed anonymously; the Niger ethics committee approved the waiver of consent

This retrospective study enrolled all children aged 5 years or less hospitalised for severe malnutrition at the intensive therapeutic feeding centre (CRENI) of the Niamey national hospital (NNH) paediatric ward (Niamey, Niger). The study period was defined as January 1^st^ 2008 to June 30^th^ 2009 as it was expected to enable the enrolment of the number of children required to accurately estimate HIV-prevalence. Under the assumption that prevalence was 5%, to obtain a precision of +/−2% with a type-1 risk of 5%, the number of children required was 457 (Nquery). Assuming a 90% acceptance of the HIV-test, 508 children were to be enrolled in the study.

For all children hospitalised for severe malnutrition during this period, information was extracted from the clinical files and made anonymous by using a unique identifier code and then filled in a standardised form. These forms were then recorded in a computerised database (Epi Info).

According to the national protocol and WHO recommendations, acute severe malnutrition was defined based on clinical signs (<70% weight for height or oedema of both feet and clinical signs of severe malnutrition) [5]. Clinical differential diagnosis was made between marasmus (global intake deficiency) characterized by severe and visible loss of weight and kwashiorkor (type 2 nutriment deficiency) mostly characterized by bilateral limb oedema. Some children may present both types of malnutrition. Finally, in children ≤6 months old, malnutrition was qualified as denutrition.

According to the national protocol, intensive renutrition lasts approximately 3 weeks in three phases: (i) acute phase of renutrition during which children, receive therapeutic milk F75 and lasts approximately 1 week; (ii) stabilisation phase during which children receive therapeutic milk F100 usually lasts around 3 days; (iii) consolidation phase of renutrition during which children receive therapeutic milk F100 and ready-to-use therapeutic food (RUTF) such as Plumpy'nut [6] during about 10 days. F75 and F100 are standardized therapeutic milks, specifically designed to treat severe malnutrition, and providing 75 and 100 kilo-calories per 100 ml, respectively [6]. Condition for discharge to the ambulatory nutrition rehabilitation unit (CRENA) was achieving a weight >85% of the weight-for-height standard in children with Marasmus or absence of complication in children with kwashiorkor.

HIV testing of the child was proposed routinely to all mothers who could accept or refuse the test. From January 2007, in children less than 18 months of age, amplification of the integrated viral genome by PCR was used to define the HIV status. HIV-positive children were referred to the HIV ward and were taken care of for free as proposed by the national programme.

Children's characteristics were compared by HIV status using student t-test for continuous variables and chi-2 test for categorical variables. Factors associated with HIV status were identified using a univariate and then multivariate logistic regression model.

Duration of hospitalisation for renutrition was defined as the duration from the date of hospitalisation to the date of discharge, death, transfer to another hospital or the date when they left hospital against medical advice. Duration of hospitalisation was described by HIV status using a competing risk approach based on cumulative incidence, and considering death as a competing risk, while children who were transferred or left hospital against medical advice were considered as censored [7]. We used the Cox proportional hazard model to identify factors, other than HIV status, which were associated with the duration of hospitalisation for renutrition. The proportional hazard assumption was validated for all factors investigated using the test on Schoenfeld's residuals.

Mortality during hospitalisation was described using Kaplan-Meier estimates, and was compared by HIV-status using the logrank test. Factors associated with mortality were identified using a parametric survival regression model with a log-normal distribution, as the proportional hazard assumption was not valid for some of the factors investigated.

In all multivariate analysis, factors associated with the event of interest with a p-value <0.2 in univariate analysis were entered in the multivariable model. Then, a backward procedure was applied to identify factors independently associated with the event of interest. All significance tests were two-sided and P-values less than 0.05 were considered statistically significant. All statistical analyses were performed using STATA 10 (Stata Corp., College station, Texas, USA).

## Results

From January 1^st^ 2008 to July 1^st^ 2009, 473 children were hospitalised for severe malnutrition, corresponding to a mean of 26 children per month. HIV testing for the children was proposed to all mothers or care-givers and was accepted in 467 (98.7%) children.

Of the 467 children with an HIV test result, 40 were found to be HIV-positive corresponding to an HIV prevalence of 8.6% (95% confidence interval (CI): 6.2–11.5). HIV prevalence was significantly higher in girls than in boys (11.5% [95% CI: 7.6–16.4] and 6.0% [95% CI: 3.4–9.7], respectively; p = 0.03) (Table 1) Median age at hospitalisation was 13 months and was not different by HIV status. Of these children, 208 (44%) were aged ≤12 months, 195 (42%) were aged 13 to 24 months, 50 (11%) were aged 25 to 36 months and only 14 (3%) were aged more than 36 months. No difference between HIV-positive and negative children were found regarding the mother's age and geographic origins defined as urban versus rural.

**Table 1:** Children's characteristics.

	HIV-negative	HIV-positive	*P*
	(N = 427)	(N = 40)	
**Demographic**			
Boys, N (%)	236 (55.3)	15 (37.5)	0.03
Age in months, median (IQR)	13 (9 ; 21)	16 (9 ; 24)	0.18
Age of the mother in years*, median (IQR)	26 (22 ; 30)	28 (25 ; 34)	0.10
Urban versus rural, N (%)	244 (56.9)	26 (65.0)	0.32
**Clinical**			
Type of malnutrition			0.51
Marasmus	254 (59.5)	29 (72.5)	
Kwashiorkor	52 (12.2)	3 (7.5)	
Mixed form	65 (15.2)	3 (7.5)	
Denutrition**	56 (13.1)	5 (12.5)	
Previous hospitalisation			<0.001
No	409 (95.3)	28 (70.0)	
Yes, for malnutrition	9 (2.1)	6 (15.0)	
Yes, for other reason	11 (2.6)	6 (15.0)	
% of the normal weight for the height, median (IQR)	67.0 (62.8 ; 71.0)	66.5 (63.6 ; 71.2)	0.50
Weight-for-age z-score***, median (IQR)	−4.7 (−5.3 ; −3.9)	−4.7 (−5.5 ; −3.9)	0.75
Height-for-age z-score, median (IQR)	−2.4 (−3.6 ; −1.5)	−3.0 (−4.1 ; −1.6)	0.23
Main other diagnosis, N (%)			
Anaemia	56 (13.1)	3 (7.5)	0.31
Pneumonia	104 (24.2)	10 (25.0)	0.93
Malaria	106 (24.7)	6 (15.0)	0.16
Gastro-enteritis	78 (18.2)	4 (10.0)	0.19

*available in 417 and 38 mothers.

**this applies to children ≤6 months of age.

***available in 423 HIV-negative and 38 HIV-positive children.

IQR: inter quartile range.

Most children (59.5% and 70.0% among the HIV-negative and HIV-positive children, respectively; p = 0.20) presented Marasmus.

The weight-for-age z-score was very low both in HIV-negative and HIV-positive children, and was not statistically different in these two groups (Table 1, p = 0.75). The z-score indicated the severe wasting (93.6% with z-score ≤−3) of this population. As expected, z-score was significantly lower in children with marasmus than in children with kwashiorkor (p<0.001).

Height-for-age z-score was also low in this population, but again not different between HIV-negative and HIV-positive children (Table 1, p = 0.23). The proportion of children being stunted (z-score ≤−2) was 64.2%, and severely stunted (z-score ≤3) was 38.7%.

A larger proportion of HIV infected children had a previous history of hospitalisation compared to HIV-negative children (30.0% versus 4.7%, respectively; p<0.001). When only previous hospitalisation for severe malnutrition was considered, the proportion remained significantly higher in HIV-positive children compared to HIV-negative children (15.0% versus 2.1%, respectively; p = 0.001).

Of the 467 children, 401 (85.9%) were referred by another hospital and 66 (14.1%) presented directly at the NNH paediatric ward (internal referral). HIV prevalence was found to be significantly higher in children from internal referral than from external referral (30.3% versus 5.0%, respectively; p<0.001).

Both in univariate and multivariate logistic regression analysis (table 2), female gender, previous hospitalisation (whether for renutrition or not) and internal referral were associated with a higher risk of HIV-positive status. None of the other factors investigated were associated with HIV status.

**Table 2:** Risk factor of HIV-positive status (univariate and multivariate analysis).

		Crude OR (95% CI)	P	Adjusted OR (95% CI)	P
Gender	Male	1	0.03	1	0.02
	Female	2.06 (1.06–4.02)		2.48 (1.17–5.25)	
Age, months	≤12	1	0.49		
	13–24	1.15 (0.56–2.33)			
	25–36	1.04 (0.33–3.27)			
	>36	3.27 (0.82–12.94)			
Age of the mother*, years	≤22	1	0.35		
	23–27	1.58 (0.56–4.50)			
	28–30	2.12 (0.74–6.06)			
	>30	2.31 (0.84–6.38)			
Reference	External	1	<0.001	1	<0.001
	Internal	8.28 (4.15–16.53)		7.90 (3.79–16.48)	
Geographical origin	Urban	1	0.32		
	Rural	0.71 (0.36–1.40)			
Previous hospitalization	No	1	<0.001	1	<0.001
	Yes, not for malnutrition	7.93 (2.73–23.02)		7.10 (2.24–22.51)	
	Yes, for malnutrition	9.69 (3.22–26.16)		8.68 (2.60–28.97)	
Type of malnutrition	Marasmus	1	0.46		
	Kwashiorkor	0.70 (0.23–2.07)			
	Mixed form	0.42 (0.12–1.42)			
	Denutrition	0.81 (0.30–2.19)			
Brachial perimeter<110 mm	No	1	0.11		
	Yes	0.49 (0.25–0.99)			
	Missing	1.21 (0.24–6.03)			
Oedema of the feet	No	1	0.11		
	Yes	0.52 (0.22–1.21)			
Weight-for-age z-score	≤−5	0.74 (0.19–2.80)	0.90		
	−4 and −3	0.82 (0.23–2.89)			
	≥−2	1			
Height-for-age z-score	≤−3	1.27 (0.49–3.32)	0.67		
	−2	0.78 (0.26–2.35)			
	−1	0.85 (0.27–2.65)			
	≥0	1			
Weight-for-height z-score	≤−4	0.53 (0.23–1.22)	0.15		
	≥−3	1			
Malaria	No	1	0.15		
	Yes	0.53 (0.22–1.31)			

*based on the 25^th^, 50^th^ and 75^th^ percentiles.

OR: odds ratio; CI: confidence interval.

**Figure 1:**
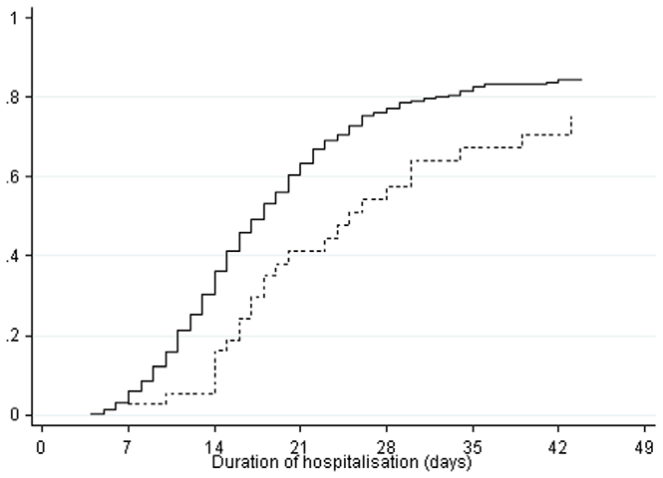
Probability of discharge to the ambulatory nutrition rehabilitation unit (CRENA) in HIV-negative (plain line) and HIV-positive (dashed line) children (competing risk approach).

Of the 467 children, 363 (76.7%) were discharged to the ambulatory nutrition rehabilitation unit (CRENA), 79 (16.7%) died during renutrition, 26 (5.5%) left hospital against medical advice, and 5 (1.1%) were transferred to another hospital. Duration of hospitalisation was longer in HIV-positive children than in HIV-negative children (Figure 1). The probability of discharge to the CRENA at 3 weeks was 63% in HIV-negative children and 41% in HIV-positive children

In a univariate Cox model all the factors presented in the table 2 were investigated. The rate of discharge to the CRENA was significantly lower in HIV-positive children when compared to HIV-negative children (crude hazard ratio (HR) [95% CI]: 0.44 [0.30–0.67]; p<0.001). It was consistent with the fact that the proportion of children who returned to phase 1 renutrition was larger in HIV-positive children than in HIV-negative children (12.5% versus 3.0%, p = 0.03). Among all other factors investigated, only gastro-enteritis diagnosed at hospitalisation was associated with a higher rate of discharge to the CRENA. The rate of discharge being higher in children who presented this condition than in the children not presenting this condition (crude HR [95% CI]: 1.52 [1.17–1.98]; p<0.001). In multivariate analysis, HIV status and gastro-enteritis condition remained independently associated with the rate of discharge to the CRENA (adjusted HR [95% CI]: 0.44 [0.29–0.65] and 1.54 [1.19–2.01], respectively; both p<0.001).

Based on Kaplan-Meier estimates (figure not shown), mortality was not statistically different between HIV-positive and HIV-negative children (p = 0.97). All factors presented in the table 2 were investigated regarding their effect on mortality. The only factor significantly associated with mortality was a malaria diagnostic, the risk of dying for more than 5-fold higher in children with malaria than in children without (crude HR [95% CI]; 5.48 [1.67–17.98]; p = 0.003).

Only considering those children who were discharged to the CRENA, both HIV-negative and HIV-positive children gained weight (in median +17.0% and +20.4%, respectively; p = 0.92). Nevertheless, weight-for-age z-scores remained low in both groups.

The most common pathologies associated with malnutrition were pneumonia in 114 (24.3%) children, malaria in 112 (23.9%) children, and gastro-enteritis in 82 (17.5%) children (Table 1), and were as frequent in HIV-negative as in HIV-positive children (p = 0.93, p = 0.16, p = 0.19, respectively).

## Discussion

This is one of the very few studies to estimate HIV prevalence in children hospitalised for acute severe malnutrition in West Africa. Indeed, a study in Nigeria reported in 1997 a HIV prevalence of 1.9% [8] while a study in Burkina Faso in 1993 reported a HIV prevalence of 14.0% [9]. Routine HIV testing of children hospitalised for severe malnutrition was implemented in 2006 at the NNH, and these results show an extremely high level of acceptance. Indeed, nearly 99% of the mothers accepted HIV testing for their children.

In this study, based on an exhaustive sample of 469 children hospitalised for acute severe malnutrition, HIV-prevalence was 8.5%. In Niger, no data on HIV-prevalence in children are available; HIV prevalence in adults is estimated at 0.8% [10], but in this population of children hospitalised for severe malnutrition, the HIV prevalence is nearly 10 fold higher. Thus, it is no longer possible to ignore that children hospitalised for severe malnutrition are a very high-risk population for HIV infection and our result pleads for implementation of routine HIV testing in nutrition rehabilitation units, as proposed by another recent study [11].

HIV prevalence was found to be significantly higher in children from internal referral (coming from the NNH) when compared to children from external referral (referred from a lower level health care facility). This result could suggest a selection bias due to the availability of a HIV-care centre in the HNN. However, it first should be noted that children from external reference represented 86% of our study population, and HIV-prevalence was 5% in these children, i.e. still around 7-fold that observed in adults. Secondly, admission in the CRENI was strictly based on nutritional status, whatever the origin of the children (internal or external reference), so that nearly no child should have been hospitalised in the renutrition unit on the basis of its HIV-status. Thirdly, following the recommended referral procedure of the gradual care level pyramid (from primary to tertiary structures) the children suffering from severe malnutrition first seen in a lower health care facility should be referred to the CRENI at the NNH which is the only renutrition unit in the region of Niamey so that we believe that this study is based on a representative set of the children diagnosed with severe malnutrition in the region of Niamey. On the other hand, the difference in HIV-prevalence between children from internal and external reference may partly result from the different geographical origins. The proportion of children from urban origins was 70% and 55% for internal and external referral respectively, and HIV prevalence is known to be higher in urban settings than in rural settings [12]. Also, one can not exclude a higher mortality in HIV-positive children during the referral delay leading to a lower HIV-prevalence in externally referred children.

Measurement errors or misinterpretation of clinical symptoms (e.g. dehydration in children suffering from gastro-enteritis) may have induced misclassification leading to the hospitalisation for renutrition of children suffering from moderate malnutrition. On the other hand, it is very unlikely that children really suffering from severe malnutrition were not identified as such and were not hospitalized. However, we believe the possible misclassification to be independent of the HIV-status, as the absence of association between gastro-enteritis condition and HIV-status suggests. Therefore, enrolment of less severely malnourished children is expected to have a very modest impact on our results.

Thus, we believe that the estimated HIV-prevalence we found (between 6.2% and 11.5% based on the 95% CI) can be generalised to the population of children hospitalized for severe malnutrition in urban referral centres in settings with low-to-middle HIV-prevalence in adults. This data also strongly suggest expanding routine HIV-testing to any stunted hospitalized child, if not to all paediatric in-patient of tertiary referral centres.

Overall, very little baseline differences were seen between HIV-positive and HIV-negative children. The limitation to this evaluation lies in the fact that this is a retrospective study based on medical records only, as used in routine care. But then, there were no demographical or clinical differences for the available parameters (table 1), except for the proportion of boys found to be lower in HIV-positive children. This lower proportion is difficult to explain as gender does not seem to increase the risk of death in paediatric cohorts [13], [14], [15]. Moreover, HIV-positive children were more likely to have had a previous history of hospitalisation. The same results were observed when only children from external referral were considered. They show how difficult it is to suspect HIV infection in the context of severe malnutrition on clinical arguments only, which pleads for a systematic screening.

About 16% of the children hospitalised for severe malnutrition died during the rehabilitation. It is important to notice that all the children benefited from cotrimoxazole prophylactic therapy. Although we did not see a higher mortality rate in HIV-positive children than in HIV-negative children, our study was limited to the duration of hospitalisation in the CRENI and children who left the hospital to continue renutrition as outpatients still presented extremely low weight-for-age z-scores and thus remain at high risk of death. Other studies with a longer follow-up have found a higher mortality in HIV-positive children than in HIV-negative children [16]. Further studies in ambulatory nutrition rehabilitation units (CRENA) are required to assess real HIV infection burden throughout renutrition and global HIV prevalence in a situation of malnutrition or a failure to thrive. According to the national protocol, the intensive phase of renutrition in CRENI is supposed to last about 3 weeks. We show that the proportion of children in whom the duration of renutrition exceeded 3 weeks is larger in HIV-positive than in HIV-negative children. Thus, HIV-infection seems to curb nutritional recovery. Since HIV-positive children were more likely to have been previously hospitalised for severe malnutrition, HIV infection is also probably responsible for a higher rate of malnutrition relapse. So far, HIV testing in nutrition rehabilitation units would not only allow a high diagnosis rate and may elucidate renutrition failures and give the opportunity of specific intervention to improve nutritional recovery. The acceptability of such a screening policy is attested by the very high test-acceptance we observed and the absence of difference in terms of early termination of nutritional care between HIV negative and positive children Essentially, it would open the door to the elaboration of specific renutrition guidelines for HIV infected children and to the questioning of the best timing for antiretroviral treatment introduction. Until now, HIV-positive children did not initiate antiretroviral therapy during renutrition but were referred to the HIV centre after discharge from the CRENI. Indeed, an earlier introduction of antiretroviral therapy could be considered, just after the stabilisation phase, to be synergistic with further nutritional care.
